# The urine biomarker panel [IGFBP7]x[TIMP-2] (NephroCheck^®^ parameter) does not correlate with IGFBP7 and TIMP-2 gene expression in urinary sediment

**DOI:** 10.1371/journal.pone.0188316

**Published:** 2017-11-16

**Authors:** Daniela Knafl, Markus Müller, Sahra Pajenda, Zeynep Genc, Manfred Hecking, Ludwig Wagner

**Affiliations:** 1 Department of Internal Medicine I, Division of Infectious Diseases and Tropical Medicine, Medical University of Vienna, Vienna, Austria; 2 Department of Internal Medicine II, Division of Angiology, Medical University of Vienna, Vienna, Austria; 3 Department of Internal Medicine III, Division of Nephrology and Dialysis, Medical University of Vienna, Vienna, Austria; University of Sao Paulo Medical School, BRAZIL

## Abstract

**Background:**

Acute kidney injury (AKI) is frequently observed in serious infections, following nephrotoxic medication, surgery and trauma. Here we tested whether the detection of two recently identified biomarkers for AKI, Tissue Inhibitor of Metalloproteinase-2 (TIMP-2) and Insulin-Like Growth Factor Binding Protein 7 (IGFBP7), depends on the expression of these proteins in cells of the urinary sediment.

**Method:**

We collected urine samples of 33 kidney transplant recipients and 14 non-transplanted patients who all had AKI (stages 1–3 according to KDIGO), and measured [IGFBP7]x[TIMP-2] using the NephroCheck^®^ Astute1 40 ^™^ meter. Concomitantly, we analyzed IGFBP7 and TIMP-2 mRNA expression by quantitative polymerase chain reaction (qPCR) from urinary sediment of the same patients, and correlated the results with [IGFBP7]x[TIMP-2] (protein), by linear regression analysis. We also determined the association between [IGFBP7]x[TIMP-2] and estimated glomerular filtration rate (eGFR), and between IGFBP7 and TIMP-2 mRNA expression and markers of inflammation. Light microscopy and confocal immunofluorescence served to illustrate changes in the urinary sediment over the time course of renal function improvement.

**Results:**

Of the 47 analyzed AKI patients, 14 presented with ascending urinary tract infection. Serum creatinine (sCr), blood urea nitrogen (BUN) and eGFR in all patients were 3.9±2.28 mg/dL, 47.59±23.1 mg/dL and 22.88±16.0 mL/min/1.73m^2^, respectively, on average ±standard deviation. [IGFBP7]x[TIMP-2] was 2.33±9.95 (ng/ml)^2^/1000, and did not associate with IGFBP7 and TIMP-2 gene expression (r = -0.0220, p = 0.4216; respectively r = 0.0972, p = 0.1909). [IGFBP7]x[TIMP-2] did not associate with eGFR; IGFBP7 and TIMP-2 mRNA expression. Improvement of renal function went along with disappearance of casts, decrease in aquaporin1 positive renal epithelial cells and leukocytes from the urinary sediment.

**Conclusion:**

The gene expression pattern of IGFBP7 and TIMP-2 from urinary sediment, which contains desquamated renal tubular epithelial cells, did not correlate with [IGFBP7]x[TIMP-2] protein, indicating that IGFBP7 and TIMP-2 measured in the NephroCheck^®^ test originated predominantly from intact but stressed cells of the kidney itself.

## Introduction

Acute kidney injury (AKI) is a frequent complication of serious infections, nephrotoxic medication, cardiothoracic surgery or trauma. Mortality rate is high due to electrolyte imbalance, overhydration and cardiopulmonary decompensation. Even minor stages of AKI can lead to chronic kidney disease (CKD), eventually requiring renal replacement therapy [1,2]. Therefore, early identification of patients at high risk for AKI is reasonable. Although serum creatinine (sCr) is considered a standard tool in clinical routine, it is not suitable for the early recognition of AKI due to inherent methodological problems [3]. AKI biomarkers such as Neutrophil Gelatinase- Associated Lipocalin (NGAL), Kidney Injury Molecule-1 (KIM-1), Cystatin C and Interleukin 18 (IL18), which are more sensitive than sCr, have been developed within the last decade [4,5].

More recently the Insulin-Like Growth Factor Binding Protein 7 (IGFBP7) together with Tissue Inhibitor of Metalloproteinases-2 (TIMP-2) were introduced as clinically highly sensitive and specific AKI biomarkers. These proteins can be measured using the NephroCheck^®^ testing method, which has been validated as a useful diagnostic tool for AKI in various clinical setting [6,7,8,9]. Importantly, NephroCheck^®^ might identify patients at increased risk of AKI, as demonstrated in a prospective observational international study with a composite endpoint of death and renal replacement therapy [10]. NephroCheck^®^ was also compared to sCr in various clinical settings, leading to AKI (e.g. kidney transplantation, cardiac failure and nephrotoxic chemotherapy [11]), and was found to differentiate between transient and permanent AKI [12,13]. In the latter three studies, NephroCheck^®^ outperformed established markers in the early identification of AKI variants [11,12,13]. Mechanistically, NephroCheck^®^‘s diagnostic potential was ascribed to the cell cycle arrest property of the analyzed proteins IGFBP7 and TIMP-2 [8,14]. Both proteins are expressed intracellularly by renal tubule cells and seem to get released as a result of tubular epithelial stress. The functions of these proteins are numerous [15], but their influence on G1-phase arrest seems to be of crucial relevance in the development of AKI.

During AKI, the composition of the urinary sediment changes, and more cellular fragments [16], kidney epithelial cells as well as leukocytes appear in the urine. This phenomenon is especially prominent in renal transplant recipients and raises the question whether IGFBP7 and TIMP-2, perhaps leaking out from desquamated cells, might significantly influence the NephroCheck^®^ parameter, or else are irrelevant. Quantitative measurement of cellular proteins derived from urine sediment is methodologically impervious. For this reason, we compared the soluble IGFBP7 and TIMP-2, which are measured by the NephroCheck^®^ test, with the gene expression in urinary sediment, aiming at elucidating the origin of the NephroCheck^®^ test proteins. Specifically, we hypothesized that the relationship between the gene expression pattern of TIMP-2/IGFBP7 and the actual concentration of these proteins in urine might allow to differentiate between protein leakage from urinary cells (resulting in a positive correlation between gene expression and protein concentration) versus release from kidney tubular cells *in situ* (and thus no correlation between gene expression and protein concentration).

## Methods

### Study population

The study was approved by the Ethics committee of the Medical University of Vienna (EK # 1598/2013 and 1043/2016). From October 2014 through March 2017, patients with AKI who were admitted to the Nephrology ward of the Medicine Department III at the Medical University of Vienna were randomly included on a continuous basis. Informed oral and written consent was obtained from each participant. Renal transplant recipients -roughly two thirds of all patients treated at our Nephrology Department were also included. AKI stage was defined according to Kidney Disease Improving Global Outcomes (KDIGO) criteria: stage 1 as a 1.5-fold increase in sCr, respective to the basal level (or an increase of more than 0.3 mg/dl); stage 2 as doubling of sCr; stage 3 as anuria or a threefold increase in sCr. Entirely anuric patients and patients with human immunodeficiency virus (HIV) or Influenza virus infection were excluded from the study.

### NephroCheck^®^ measurement

Urine of kidney transplant recipients was obtained from a Foley catheter, urine of non-transplant patients was acquired either by Foley catheter or spontaneous urination. The urine was centrifuged at 3000 rpm for 5 minutes in an Allegra^®^ X-12R Centrifuge (Beckman Coulter^®^). For NephroCheck^®^ measurement, 100 μl precleared urine together with 100 μl NephroCheck^®^ buffer were mixed in the NephroCheck^®^ conjugate vial, as described in the test manual. The loaded NephroCheck^®^ cartridge was inserted into the NephroCheck^®^ test meter Astute140^®^, and after 20 minutes the numeric value of AKI risk score (AKRS), resulting from the calculated product [IGFBP7]x[TIMP-2] in (ng/ml)^2^/1000 (referred to as NC-score), was recorded. At the same time the same patients’ urinary sediment was lysed in Trizol media and frozen at -20°C for subsequent RNA extraction. In addition an aliquot of cell suspension was used for cytospin preparation.

### Quantitative PCR

RNA was extracted by mixing the previously frozen Trizol lysate with chlorophorm and precipitating the total RNA using isopropanol, as described in the Trizol test manual. Purified RNA was dissolved in RNAse free water and mixed with dNTPs random hexamer primers and reverse transcriptase using superscript enzyme. The resulting cDNA was diluted 1:4 with H_2_O and amplified using IGFBP7 and TIMP-2 specific probes from TaqMan^®^ (Thermo Fisher Scientific) and 2x TaqMan^®^ Universal Master Mix in a StepOnePlus qPCR machine (Applied Biosystems^®^). Data recording was performed over 46 cycles. Individual IGFBP7 and TIMP-2 expression levels in terms of cycle threshold (Ct) were normalized using GAPDH as house keeping gene resulting in a ΔCt value, and further calculated using the ΔΔCt method [17]. The expression level of normal human kidneys obtained from tumor nephrectomy was chosen as reference.

### Confocal immunofluorescence

Cytospin preparations were prepared from cells derived from 10 ml urine, centrifuged at 3000 rpm for 5 min. The cell pallet was suspended in 2500 μl tissue culture media, and 100 μl cell suspension was applied into the Shandon cyto-centrifuge funnel. Following air-drying for 2 hours, the slides were either frozen, wrapped into aluminum foil, or immediately processed for Hematoxylin-Eosin (HE) staining. For immunofluorescence staining the slides were fixed in acetone and wetted with phosphate buffered saline (PBS) followed by incubation with primary antibody diluted 1:100 (mouse-monoclonal-antibody against TIMP-2, Santa Cruz sc-21735) and rabbit anti human AQP1 diluted 1:800 (Millipore AB 2219) overnight at 4°C and the next day with Alexa Fluor^®^ 488 goat anti rabbit (diluted 1:400) and TRIC goat anti mouse (diluted 1:400) for one hour at room temperature. Each step of antibody staining was followed by PBS washes under constant stirring. 4′,6-diamidino-2-phenylindole (DAPI) was applied during the last minutes of secondary antibody incubation. Slides were mounted in Vectashield^™^ and viewed under a Zeiss^®^ confocal microscope and images were further processed using Photoshop^®^ version 6 for Microsoft^®^.

### Calculations and statistics

Analysis of both, the mRNA expression patterns of IGFBP7 and TIMP-2 in urinary sediment, and the NephroCheck^®^ measurement, were carried out on blinded samples. The numeric values of the [IGFBP7]x[TIMP-2] in (ng/ml)^2^/1000 were correlated with the relative expression level of IGFBP7 and TIMP-2, respectively. The mean of 3 tumor nephrectomies was taken as reference level. The correlation coefficients were calculated using the Pearson square method with Graph Pad Prism^®^.

#### Sample size calculation and power analysis

Sample size was calculated to test for a correlation coefficient of *r ≥ 0*.*4*, consistent with a moderate to strong correlation, with a power of *80%* at a significance level of *α = 0*.*05*. According to our calculations, a total of at least *n = 37* patients needed to be included in order to meet the tended criteria. Based on a one tailed test, the null hypothesis (*H0*: *0 ≤ r < 0*.*4*) was rejected if the correlation coefficients of [IGFBP7]x[TIMP-2] and IGFBP7 gene expression, as well as [IGFBP7]x[TIMP-2] and TIMP-2 gene expression exceeded a critical correlation coefficient of *r = 0*.*28* respectively, with an *α* of *0*.*05* and a *β* of *0*.*2*. Since this is a non-interventional study, patients who met the inclusion criteria were enrolled on a continuous basis after the required sample size was already achieved. This was done to further increase statistical power and validity.

A total of 47 patients (*n = 47*) were enrolled and included in final analysis. The study was powered to detect a correlation coefficient of *r ≥ 0*.*35* with a power of *80%* at a significance level of *α = 0*.*05*, with a critical correlation coefficient of *r = 0*.*24*.

## Results

The study population consisted of 47 adult patients with newly diagnosed AKI admitted to the nephrology ward of the General Hospital of Vienna between October 2014 and March 2017. 33 patients had received a kidney transplant in the past and 14 patients were non-transplant recipients (Tables 1 and 2). Reasons for AKI among the 33 transplant recipients were ascending urinary tract infections (UTI) in fourteen patients, eleven were experiencing delayed graft function (DGF), acute kidney transplant rejection in three patients, hypovolemic shock in one patient and low fluid intake in one patient, also suffering from a wound infection and three patients were shortly after kidney transplantation (Tables 1 and 2). Thirteen patients experienced AKI stage 1, seven patients had AKI stage 2 and thirteen patients suffered from AKI stage 3. Two patients required intermittent haemodialysis.

**Table 1:** Demographics and laboratory values.

	All	Normal Range
Number of Patient (%)	47 (100)	
Transplant recipients (%)	33 (70)	
Female (%)	17 (36)	
Age [years]	59±16	
AKI stage I (%)	15 (32)	
Transplant recipients	13	
AKI stage II (%)	13 (27)	
Transplant recipients	7	
AKI stage III (%)	19 (40)	
Transplant recipients	13	
Patients requiring haemodialysis (%)	3 (6)	
Transplant recipients	2	
sCr [mg/dl]	3.9±2.28	0.5–0.9
eGFR [ml/min/1.73m^2^]	22.88±16.1	>90
NephroCheck^®^ score [(ng/ml)^2^/1000]	2.33±9.95	
CRP [mg/dl]	6.15±7.7	<0.5

Categorical variables are reported as counts and frequencies. Continuous variables are reported as medians and ranges, or means ± standard deviations.

**Table 2:** Table of confounders and individual patients´ demographics.

ID	Transplant recipient	Sex	Age in years	AKI related diseases	Microbiology	KDIGO stage
1	yes	M	40	Rejection episode		2
2	yes	M	52	Wound infection, hematoma	ni	1
3	yes	M	46	DGF		3
4	yes	M	71	Kidney transplant		1
5	yes	M	55	Kidney transplant		1
6	yes	M	77	DGF		3
7	no	M	68	Infection, UTI	*E*. *coli*	3
8	no	F	76	Crush syndrome, rhabdomyolysis		3
9	yes	M	35	DGF		3
10	yes	M	62	DGF		3
11	no	M	24	HUS	ni	2
12	yes	M	48	DGF		3
13	yes	M	36	Infection, PE	ni	1
14	yes	F	53	DGF		3
15	yes	F	67	Rejection episode		3
16	yes	M	57	DGF		3
17	yes	M	77	DGF		3
18	no	M	74	Infection, UTI	ni	2
19	no	F	61	Infection, UTI	ni	3
20	yes	M	28	Infection, UTI	*P*. *aeruginosa*	1
21	no	F	53	Chemotherapy		3
22	yes	F	77	Infection, UTI	ni	1
23	yes	M	62	DGF		3
24	yes	F	69	Infection, UTI	*E*. *coli*	2
25	no	F	46	Sepsis, undefined focus		3
26	yes	F	27	Infection, UTI	*E*. *coli*	1
27	no	F	34	Sepsis, cholecystitis	ni	1
28	no	M	51	Cardiac failure, NYHA IV		2
29	yes	F	79	Kidney transplant		1
30	yes	M	61	Infection, UTI	*E*. *coli*	3
31	yes	F	60	Infection, UTI	ni	1
32	no	F	63	Cardiac failure, NYHA IV		2
33	yes	M	76	DGF		3
34	yes	M	56	Infection, UTI	*E*. *coli*	1
35	yes	M	70	Infection, UTI	ni	1
36	no	M	62	Cardiac failure, NYHA IV		2
37	yes	F	95	Infection, UTI	*E*. *coli*	2
38	yes	M	22	Infection, UTI	*E*. *coli*	2
39	no	M	60	Chemotherapy, pheochromocytoma		3
40	yes	M	76	Rejection episode		2
41	yes	M	47	Infection, UTI	*E*. *coli*	2
42	yes	M	56	Infection, UTI	ni	2
43	no	F	61	Sepsis, undefined focus		1
44	no	M	58	Chemotherapy		2
45	yes	M	39	Infection, UTI	*E*. *coli*	1
46	yes	F	67	Infection, UTI	*E*. *coli*	1
47	yes	F	78	DGF		3

Individual patient data: transplant status, gender, age, underlying disease, isolated bacteria, stage of AKI according to KDIGO

Delayed graft function (DGF), not identified (ni), urinary tract infection (UTI), pulmonary embolism (PE), New York Heart Association IV (NYHA IV) defined as dyspnea due to cardiac failure while patient is at rest, *Escherichia coli* (*E*. *coli*), *Pseudomonas aeruginosa* (*P*. *aeruginosa*).

Among the 14 non-transplant recipients, three suffered from chemotherapy-induced AKI, three experienced AKI due to sepsis, another three due to ascending urinary tract infection, three were undergoing cardiac failure, one was diagnosed with hemolytic uremic syndrome and one experienced a crush syndrome with rhabdomyolysis. Two patients were assigned to AKI stage 1, six patients to AKI stage 2, and six patients to AKI stage 3. One patient required haemodialysis and eventually developed end-stage renal disease (ESRD) (Tables 1–3).

**Table 3:** Underlying diseases most likely causing AKI.

**renal transplant recipient**	*n*
*cause of AKI*	
pyelonephritis	9
delayed graft function	10
rejection episode	2
wound infection	1
hypovolemic shock	1
kidney transplantation	3
**non transplanted**	*n*
*cause of AKI*	
chemotherapy induced kidney injury	3
rhabdomyolysis	1
sepsis	3
pyelonephritis	3
hemolytic uremic syndrome	1
cardiac failure	3

Underlying diseases most likely causing AKI in renal transplant patients (A) and patients without kidney transplantation (B).

The mean NephroCheck^®^ score at enrollment was 2.33±9.95 in (ng/ml)^2^/1000 and mean sCr was 3.9±2.28 mg/dl. The mean eGFR was 47.78±17.2 mL/min/1.73m^2^, mean C-reactive protein (CRP) was 6.15±7.74 mg/dl. Pearson square pairwise correlation analysis revealed that the NephroCheck^®^ score did not associate with IGFBP7 and TIMP-2 gene expression (r = 0.0102, p = 0.4726, and r = 0.1277, p = 0.1962 respectively).

**Fig 1:**
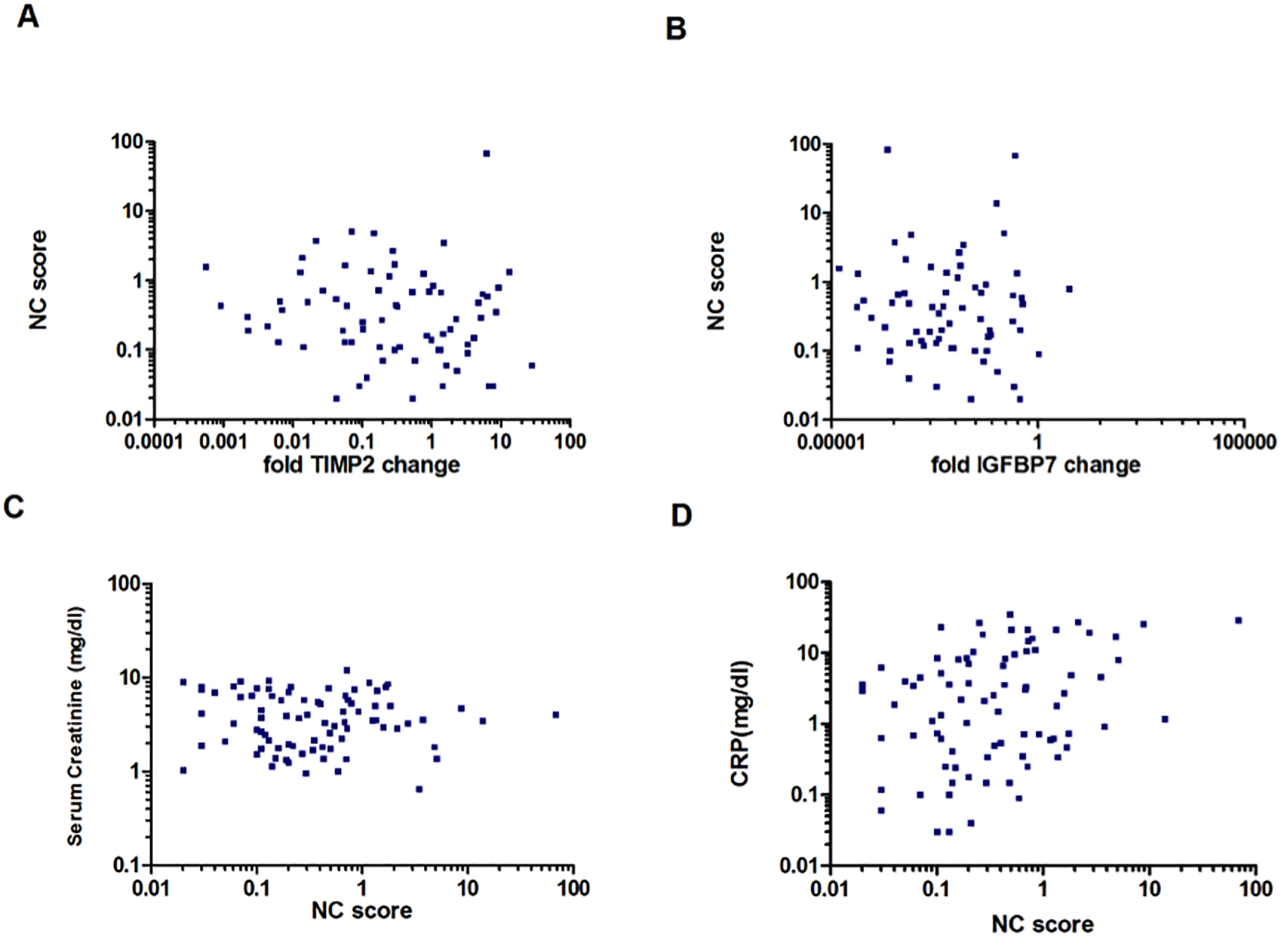
Correlation of NC-score with relative change of TIMP-2 and IGFBP7 expression, sCr and CRP. A) Pearson square pairwise correlation of NC-score with relative change of TIMP-2 mRNA expression in urinary sediment cells taking three normal kidney tissues as reference level. B) Pearson square pairwise correlation of NC-score with relative change of fold IGFBP7 change in urinary sediment cells taking three normal kidney tissues as reference level. C) Pearson square pairwise correlation of NC-score with serum creatinine levels (mg/dl) (r = -0.0283, p = 0.3995). D) Pearson square pairwise correlation of NC-score with CRP (mg/dl) (r = 0.3382, p = 0.0009).

Nine patients underwent up to 4 follow up measurements of both the NephroCheck^®^ score and the qPCR for IGFBP7 and TIMP-2 expression in urinary sediment resulting in 83 individual points of measurement. As depicted in Fig 1A and 1B there was no association between IGFBP7 and TIMP-2 mRNA expression in urinary sediment and NephroCheck^®^ score in the entire patient group (r = 0.02204, p = 0.4216; respectively r = 0.0972, p = 0.1909). The NephroCheck^®^ score did not correlate with sCr (r = -0.1786, p = 0.1684, Fig 1C) and showed marginal correlation with CRP (r = 0.338, p = 0.018, Fig 1D). Regarding the two different subgroup analyses comprising the two most frequent etiologies of AKI in enrolled transplant recipients (UTI and DGF), no significant correlations were found between NephroCheck^®^ score and IGFBP7 gene expression or between NephroCheck^®^ score and TIMP-2 gene expression. There were also no significant correlations found between the 33 transplant recipients or among the 14 non-transplant recipients.

**Fig 2:**
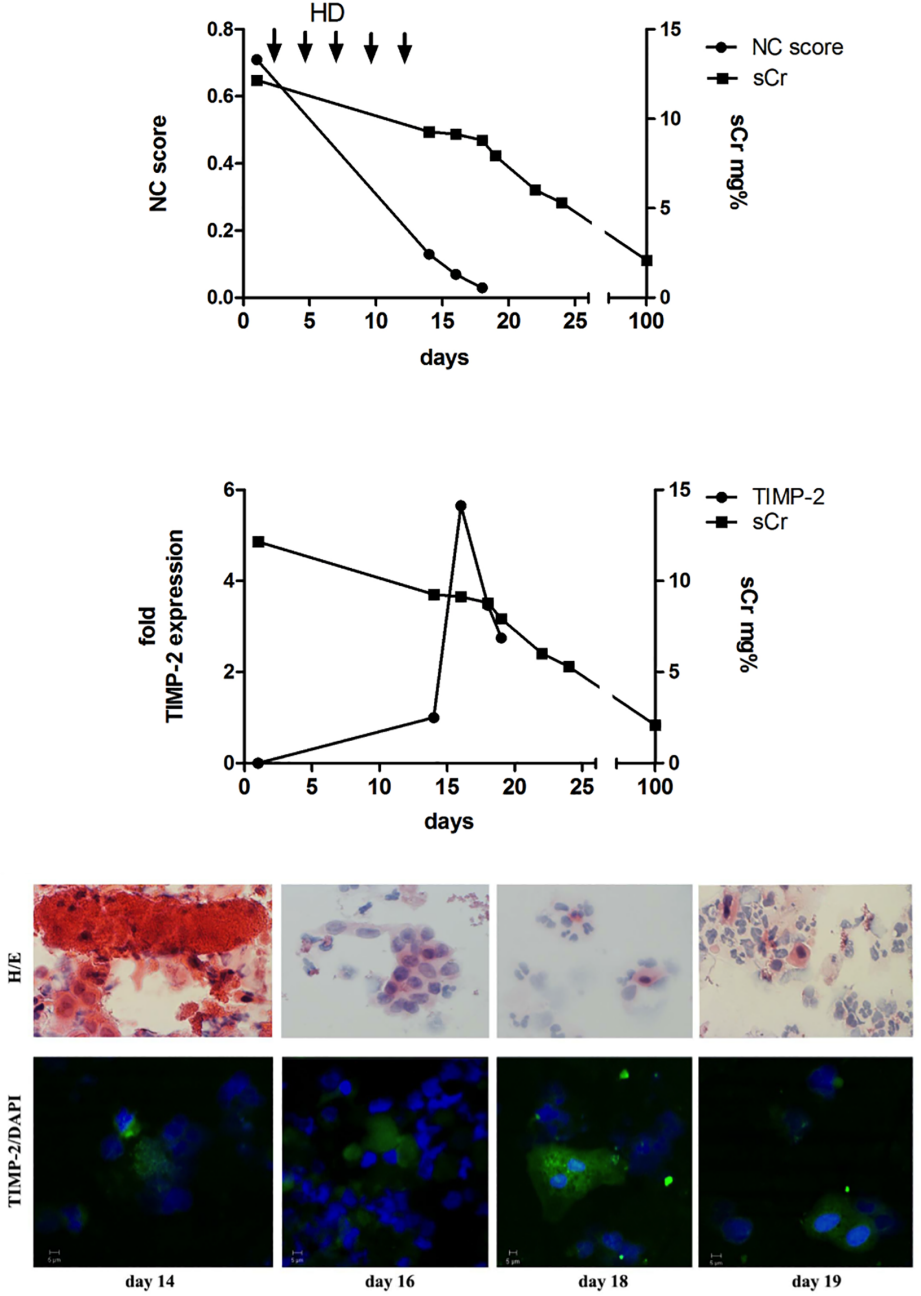
NC score, sCr, normalized fold TIMP-2 expression and staining of urinary sediment of representative patient #1. A) A representative patient after kidney transplantation, but requiring haemodialysis (HD) throughout the first week post-transplant (DGF). Improvement of renal function was accompanied by an increase of urinary sediment TIMP-2 expression, and decrease in NC score parameter. 100 days after kidney transplantation the patient was alive with a serum creatinine of 2.1 mg/dL. B) HE (upper panel) and confocal immunofluorescence staining (lower panel) of urine sediment from the representative patient with delayed graft function. Many morphologically disintegrated cells including casts appeared in urine (day 14). This changed rapidly the following days, cell number in urine increased from day 14 to day 17. Furthermore, the percentage of TIMP-2 positive staining cells increased with presence of a mitosis (day 18) while the number of disintegrated cells decreased, accompanied by rapid fall in NC-score and decrease in sCr.

HE staining and a microscopic evaluation of urine were performed in all patients. Fig 2B at the upper panel represents the course of patient#1. This patient is also depicted in Fig 2B lower panel for TIMP-2 staining. 19 patients showed casts under which 14 were transplant recipients. Leukocyturia was found in 35 patients; among them 26 were transplant recipients. Analysis of protein expression in urinary sediment using TIMP-2-specific immunostaining was performed in fourteen representative patients (Fig 2B lower panel representing patient#1) upon admission, during and after recovery from AKI. Upon admission, TIMP-2 expressing cells were found in urinary sediment among epithelial cells and leukocytes. Several of these cells had undergone signs of partial lysis such as nuclear fragmentation, loss of nucleus, membrane disintegration and cell debris formation. Strong staining was observed in cells with typical epithelial cell morphology.

**Fig 3:**
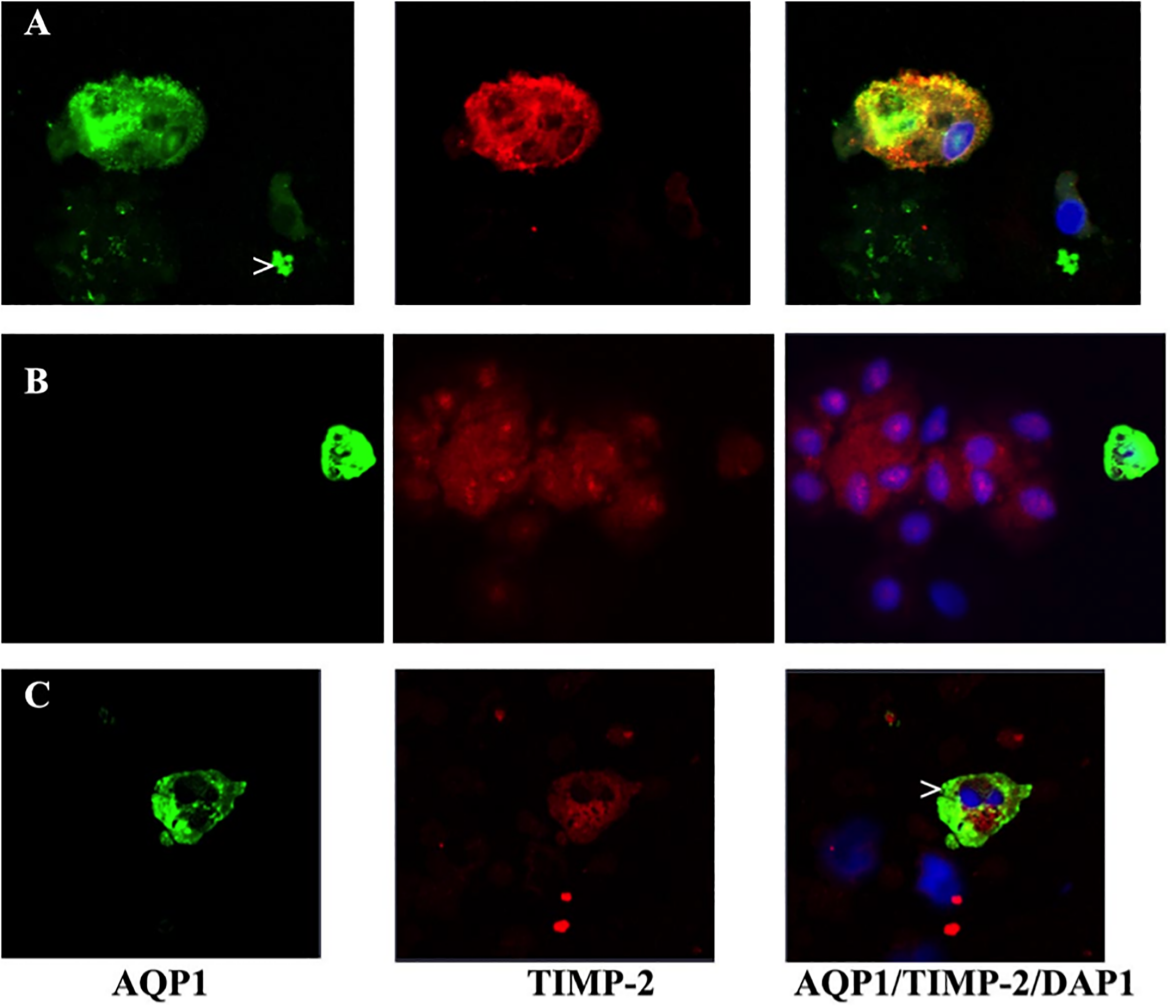
Dual colour confocal microscopy of urinary sediment. A) Kidney epithelial cell originating from the proximal tubule identified by AQP1 positive staining (green). TIMP-2 expression (red) is also present to high extent. The cell shows signs of membrane lesion and the nucleus is condensed. Cell debris (>) positive for AQP1. B) One AQP1/TIMP-2 (green/red) proximal tubule cells and TIMP-2 positive epithelial cell (red) not originating from the proximal tubule. C) AQP1/TIMP-2 (green/red) positive proximal tubule cell showing nuclear fragmentation (>) in form of apoptotic bodies from patient #2 at the time point when urine production started.

In a further attempt to identify proximal tubular epithelial cells dual colour confocal microscopy was performed on these fourteen patients. Shortly after transplantation up to 4% aquaporin 1 (AQP1) positive cells (= proximal tubular origin, green) were detected most of them positive to TIMP-2 as well (red) (Fig 3A to 3C). These cells show signs of membrane lesion (Fig 3A) or nuclear apoptosis (>) (Fig 3C) and presence of proximal tubule cell debris (>) (Fig 3A). Positive TIMP-2 cells were also seen negative for AQP1 during the phase of recovery (Fig 3B).

## Discussion

In the present study performed with AKI patients in a typical clinical setting, the NephroCheck^®^ score ([IGFBP7]x[TIMP-2]) did not associate with IGFBP7 or TIMP-2 mRNA expression in urinary sediment cells. Furthermore, there was no correlation with sCr, BUN or eGFR. Immunofluorescence staining with TIMP-2-specific immuno-reagents demonstrated presence of the protein within the cytoplasm of renal epithelial cells to some extent originating from the proximal kidney tubule as well as in a leukocyte subpopulation. Proximal kidney tubular cells were identified by dual colour confocal microscopy using AQP1 staining. The urinary sediment may be abundant especially in urinary tract infections and therefore can influence the protein content of urine. Ascending urinary tract infections are frequent among renal allograft recipients [18]. As TIMP-2 is found within the cells present in urine, it is conceivable that these cells might influence the [IGFBP7]x[TIMP-2] level. The present data emphasize the opposite, rather supporting the notion that the urine value of [IGFBP7]x[TIMP-2] originates from kidney epithelial cells *in situ* and not from other cells present in urine (e.g. urothelial cells, leukocytes, and others). A multicenter study performed by Zarbock et al. demonstrated an increase in NephroCheck^®^ score after remote ischemic preconditioning, which was indicative for the protection of study participants from AKI during coronary artery bypass operation [19]. The authors hypothesized that remote ischemic preconditioning and resultant changes in hemodynamics or humoral factors were responsible for the increase of NephroCheck^®^ score. Videlicet, a consecutive cell cycle arrest within the kidney may change the susceptibility for AKI. A similar scenario was observed by a second research group, which described a higher NephroCheck^®^ score at time point zero (H0) than four hours (H4) after a noxious event, which led to a higher delta NephroCheck^®^ score (H4-H0). Patients with higher delta NephroCheck^®^ score were more likely to undergo transient AKI in comparison to patients with low delta NephroCheck^®^ score, who were more likely to experience *persistent* AKI [12]. These results indicate that faster kinetics were associated with better prognosis and recovery of AKI [12].

Recent *in vitro* experiments demonstrated that human tubular epithelial cells are the region of origin of IGFBP7 and TIMP-2 [20]. Specifically, TIMP-2 originated from distal tubular cells and was increased in tissue culture supernatant as well as within the cells following reperfusion upon oxygen and nutrient deprivation. IGFBP7, however, was found to be located in proximal tubular cells [20]. However, it is still a matter of debate whether this holds true for *in vivo* conditions. Data from animal models are publically available in GEO profiles at NCBI; these data indicate that kidney epithelial cells up-regulate TIMP-2 upon ischemic and pharmaco-therapeutic stimuli or diet GPL1355, 1386940_at (ID_REF), GDS4812, 29543, substantiating the data described in Figs 2A and 3 that TIMP-2 expression is up-regulated days after reperfusion injury. Again, this phenomenon points towards a physiological and protective effect of the NephroCheck^®^ proteins. This is supported by the finding that urinary IGFBP7 was identified as a predictive factor indicative for good chance of recovery from AKI [21].

**Fig 4:**
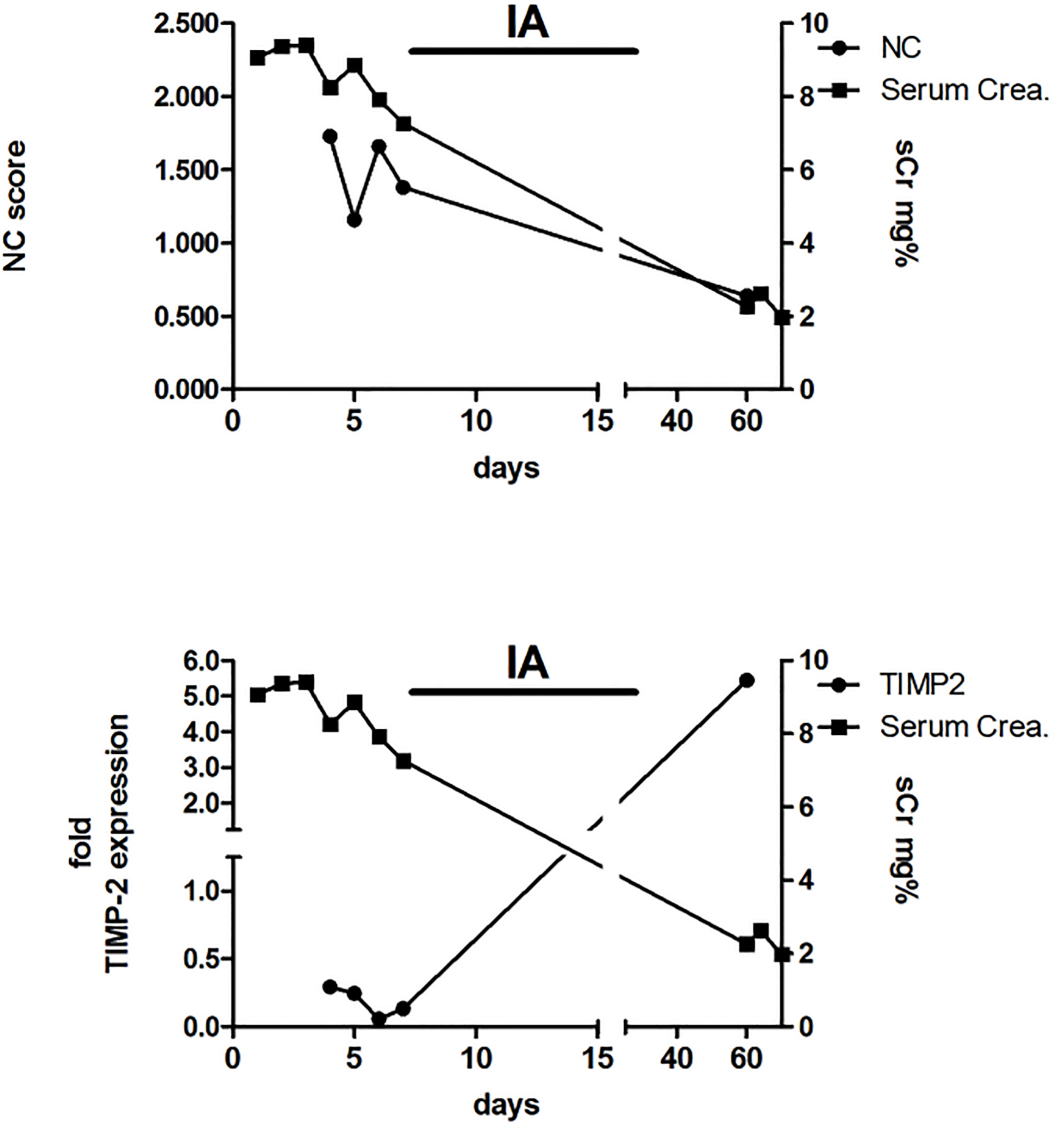
NC score, sCr and normalized fold TIMP-2 expression of representative patient #2. A 74 year old male patient having received a cadaver renal allograft from a 79-year-old donor showing delayed graft function and histologically verified signs of antibody mediated rejection. He underwent 12 sessions of immunoapharesis (IA) using a protein A column. His renal function gradually was improving and so the NC score as well as the urinary cell count and cell morphology.

The increase of TIMP-2 mRNA in urinary sediment as shown in Figs 2A and 4 during the late phase and recovery from AKI and reperfusion injury, reflects a higher responsive rate of transcription within the various stretches of the renal tubule. The desquamated tubular cells in urine originate from various stretches of the tubule and contribute to the higher amount of IGFBP7 and TIMP-2 mRNA.

In a further attempt to visualize the course of the individually measured parameters of patients with AKI, we demonstrate that the relation of TIMP-2 mRNA expression in urinary cells is rather inverse with the NephroCheck^®^ score (Figs 2A and 4) and therefore we describe their medical history as a vivid example below.

**Patient #1)** The course of the NC score, sCr and normalized fold TIMP-2 expression in the urinary sediment of a 60 year old male patient with delayed graft function is depicted in Fig 2. He received a cadaver kidney transplant from a 70 years old donor. The patient required HD throughout the first 13 days but urine production gradually improved. This was going along with an increase of TIMP-2 expression in urinary sediment while the NC score was decreasing.

**Patient #2)** A 70 years old male patient (Fig 4) received a cadaver renal transplant from a 79 years old donor. The patient required HD the first 6 days after transplantation. A kidney biopsy was taken from the upper part of the transplant which revealed signs of antibody mediated rejection. Under thirteen sessions of immunoapharesis using a protein A column the renal function (sCr) was improving going along with falling NC score. The patient was well with a sCr of 2mg/dl 60 days after transplantation. TIMP-2 expression in urinary sediment was at the highest level at 60 days post-transplant when renal function was almost recovered and urinary sediment contained morphologically intact epithelial cells with absence of AQP1 positive tubular epithelia. This has to be interpreted as increase in gene expression and intracellular protein while the NephroCheck^®^ score value is declining during recovery of kidney function. The increased expression in kidney epithelial cells following an episode of reperfusion injury, without any further noxious or traumatic stimulus inhibits further secretion of IGFBP7 and TIMP-2 in urine. This becomes apparent in the drop of NephroCheck^®^ score.

However, whether the positive effect of NephroCheck^®^ score fast kinetics are associated with increased transcription within the kidney must be left unanswered because of the limitations of this study. Further studies would have to proof this by histological means within the kidney of AKI patients. Another limitation of this study is, that it was solely performed on medical patients and cannot be expanded to surgical conditions. However, it is important for all further studies that urinary cells do not influence the urinary NephroCheck^®^ score. Whether the gene expression of IGFBP7 and TIMP-2 from the urinary sediment translates into higher protein levels in urine remains to be determined in future studies.

In conclusion, the extent and type of urinary sediment does not affect the results of [IGFBP7]x[TIMP-2]. The [IGFBP7]x[TIMP-2] values reflect the process ongoing *in situ* in the nephrons.

## Supporting information

S1 Table Fold gene expression. Itemized details of pseudonymised patients including TIMP-2 and IGFBP7 fold gene expression.
(XLSX)

## Abbreviations

AKI: acute kidney injury
AKIRisk: [IGFBP7]x[TIMP-2]
AQP1: aquaporin 1
cDNA: copy deoxyribonucleic acid
CRP: C-reactive protein
CT: cycle time
DGF: delayed graft function
eGFR: Estimated Glomerular Filtration Rate
HD: haemodialysis
IA: immunoapharesis
IGFBP7: Insulin like Grows Factor Binding Protein 7
mRNA: messenger RNA
NC: NephroCheck^®^
ni: not identified
NYHA: New York Heart Association IV
PE: pulmonary embolism
qPCR: quantitative Polymerase Chain Reaction
sCr: serum Creatinine level
TIMP-2: Tissue Inhibitor of Metalloproteinase-2
UTI: urinary tract infection
